# Different environmental requirements of female and male Siberian ibex, *Capra sibirica*

**DOI:** 10.1038/s41598-021-85550-6

**Published:** 2021-03-16

**Authors:** Lei Han, Zhi Wang, David Blank, Muyang Wang, Weikang Yang

**Affiliations:** 1 State Key Laboratory of Desert and Oasis Ecology, Xinjiang Institute of Ecology and Geography, Chinese Academy of Sciences, Urumqi, 830011 China; 2 Mori Wildlife Monitoring and Experimentation Station, Xinjiang Institute of Ecology and Geography, Chinese Academy of Sciences, Mori, 831900 China; 3 University of Chinese Academy of Sciences, Beijing, 100049 China; 4 Research Center for Ecology and Environment of Central Asia, Bishkek, 720001 Kyrgyzstan

**Keywords:** Ecology, Zoology

## Abstract

In sexually dimorphic species, males and females may select different habitat for greater fitness. However, the key factors that play a leading role between sexes in habitat selection are still poorly understood. In this paper, we investigated the possible causes of the differences in habitat preference between male and female Siberian ibex (*Capra sibirica*) living in the Tianshan Mountains (China). Using the Maximum entropy model, we found that the ruggedness and elevation of the terrain were the most important factors affecting habitat selection in Siberian ibex. Females preferred the most rugged terrain to increase the security of their young and themselves, while males favored moderately rugged terrain to provide sufficient safety from predators, and availability of suitable forage simultaneously. Females used a wider variety of elevations to search for newly emerged vegetation for its higher nutritional value, while males preferred more elevated slopes to avoid the higher temperatures and greater presence of biting insects found at the lower elevations. In addition, females were associated more with rivers due to their higher water demands. The differences in habitat selection between Siberian ibex males and females depend on multiple considerations, but only a limited number of key factors determine their actual distribution.

## Introduction

Selection of the most suitable habitat is a crucial process in the strategy for survival of animals^1^. This selection process depends on the specific environmental conditions that enable a species to prosper and reproduce successfully, therefore optimal habitat selection could result of greater fitness for both the individual and the population as a group^2^. Food acquisition and security from predators are two primary factors which determine the habitat selection in ungulates^3^, and individuals may thus select habitats that provide rich forage or reliable refuge from predators^3,4^. In habitat selection, food demands are often in conflict with security from predators, so individuals need to trade-off between these different habitat characteristics. This trade-off has been well documented in many species; for example, the Nubian ibex (*Capra nubiana*) prefers open cliff habitats for safety during spring, but change their habitat for increased forage during summer^5^.

It has been reported that not only do different species have various preferences in habitat selection, but the different sexes of conspecifics also demonstrate different preferences^6,7^. In sexually dimorphic ungulates, significant differences in body size of two sexes, leading them have distinctions in energy requirements and digestive capabilities^8,9^, differences in exposure to predation risk^10^, and different roles in the reproductive process^10^.As a result, adult males and females select different habitats all year round, only joining together during mating season^11,12^. Several hypotheses, each focuing on only one aspect of those differences, have been proposed to explain the distinctive habitat preferences in male and female ungulates^10^.

Based on the different digestive efficiency and resource requirements, some researchers proposed that, according to the Jarman-Bell principle, larger individuals are physiologically more effective in digesting lower quality forage than smaller individuals^8,9^. Smaller females therefore will select preferentially higher quality food habitats, whereas males will use lower quality but higher biomass habitats compared to females^13^. In addition, considering the different strategies used by each sex to maximize reproductive success, some researchers proposed that females and their offspring are often more vulnerable to predation risk than adult males; therefore, to maximize their and their offspring’s survival, and ultimately long-term reproductive success, females would use the habitats with a lower predation risk. While the reproductive success of males depends on their chances of access to females, which is related to the physical condition of males, so males should select habitats with forage of high quality and quantity to favor body growth, regardless of predation risk in those areas^10,14^. Furthermore, some other researchers proposed that males and females selected different habitats because of their different sensitivity to weather conditions: The larger body-sized males have a higher sensitivity to high summer temperatures than the smaller body-sized females^15^. This phenomenon is related to the different relative surface areas of the male and female bodies, where smaller females have a larger surface area to volume ratio and a greater ability to dissipate their extra body heat and avoid overheating than larger males (thermal resistance)^16,17^. Therefore, it would be expected that males would move to higher elevated areas with cooler air and stay there more often, and avoid staying in lower-elevated areas with higher summer temperatures. In contrast, females would be less sensitive to hot weather and choose to use wider elevations in summer.

The Siberian ibex (*Capra sibirica*) is distributed throughout the TianShan Mountains, Pamir, Himalaya, Karakorum, Altai, and the mountains of Southern Siberia and Mongolia^18^. It has been classified as a Category I Protected Wild Animal Species under the Wild Animal Protection Law and listed as "Endangered" in the China Red Data Book of Endangered Animals. The Siberian ibex is a sexually dimorphic species with the typical body size of a male twice as much as the size of a female’s (90 kg vs 44.2 kg)^18^. Siberian ibex can climb and move through rugged terrain more quickly than their predators and use the cliffs and rocks as a refuge^18^. Habitat selection of Siberian ibex has been studied by many authors^19–21^, but until now little attention has been paid to investigating potential differences in habitat selection between males and females, and the key factors leading to habitat segregation are still poorly understood. Identifying the most important environmental factors to habitat selection in ungulates is helpful for understanding their strategy to maximize population fitness and survival, and is also helpful in the management and conservation of ungulates^2^.

The main objective of this study was to determine the relative ranking of environmental factors that are most important for habitat selection of Siberian ibex, and investigate the divergence of Siberian ibex in habitat preference between males and females. Since Alpine ibex (*Capra ibex ibex*) females and males showed habitat segregation during summer^22^, we also expected similar behavior in Siberian ibex, to maximize survival and reproduction success, females and males would select different habitats. Considering the more vulnerability of females to predation risk compared to males^23,24^, we predicted that females would prefer more rugged terrain than males in favor of safety of their offspring and themselves. In addition, our previous observations demonstrated that males preferred the most abundant plant species that can supply the greatest volume of food irrespective of its quality, while females selected plants based on their higher nutritional content^24^. We therefore predicted that females would use a wider range of elevations to track emerging, high-quality plants, whereas males would exploit a narrower range of elevations to access a higher abundant forage irrespective of its quality. Finally, we expected that the larger body-sized males would be more sensitive to the hot climate conditions than the smaller body-sized females, because the females have a relatively larger body surface area to volume ratio compared to males and would, therefore, have a greater ability to release body heat; and in summer males would prefer to select elevated areas with lower temperatures.

## Results

### Importance permutation of habitat variables

**Figure 1:**
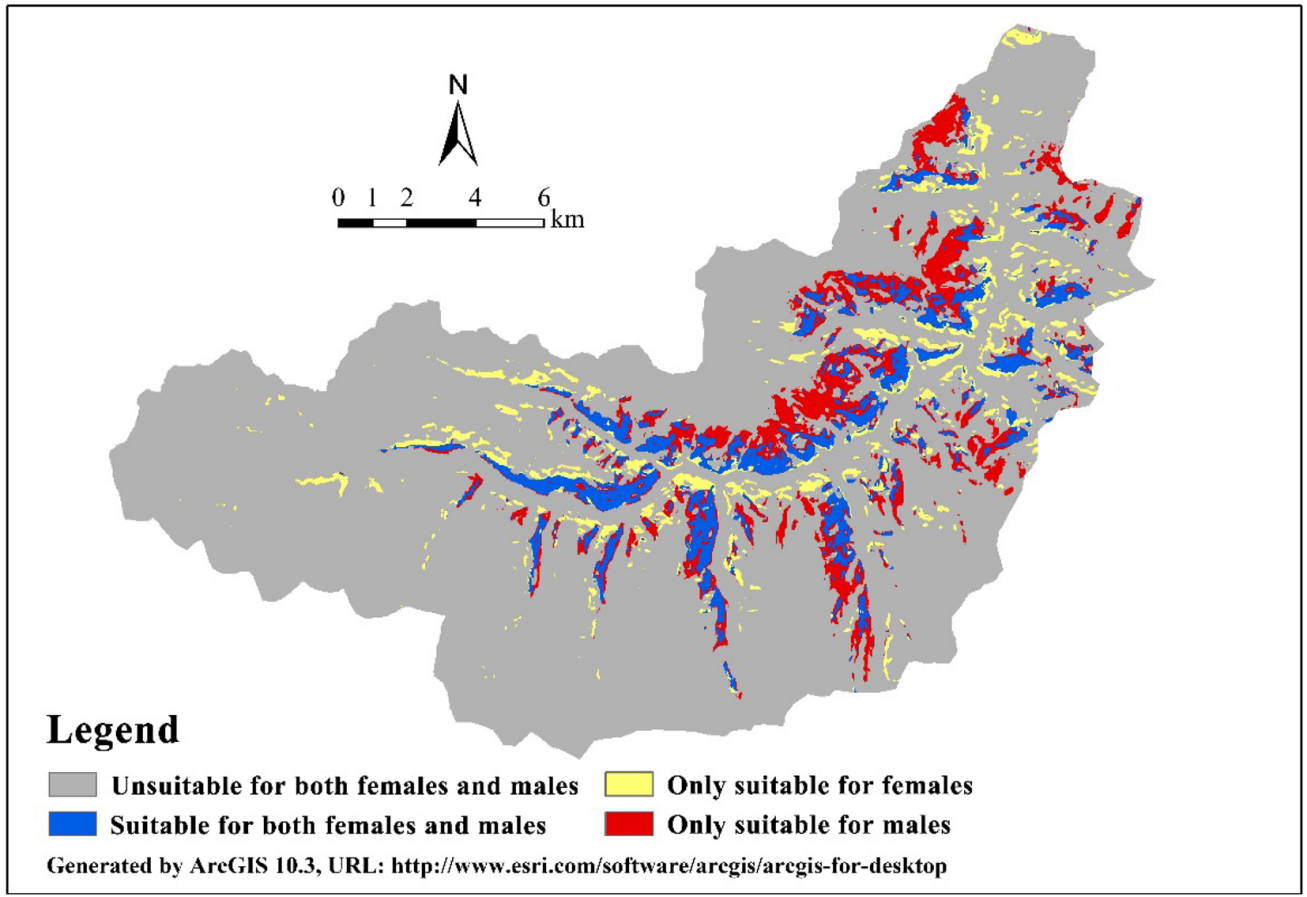
Different habitat preferences for female and male Siberian ibex.

Our results showed that the model quality of female Siberian ibex was good (AUC = 0.819, SD = 0.065), while in male Siberian ibex, it was fair (AUC = 0.787, SD = 0.114). The MST criteria of female Siberian ibex was 0.62, and male Siberian ibex was 0.58. Habitat suitability maps showed a different habitat preference between female and male Siberian ibex, with the overlap ratio on females’ and males’ suitable habitat was 35.12% (Fig. 1). The suitable habitat area of female Siberian ibex (14.21 km^2^) was smaller than males’ (26.17 km^2^), and the mean size of the suitable habitat of female Siberian ibex (0.03 km^2^) was also smaller than that of males (0.09 km^2^). Table 1 shows the importance permutation of habitat variables of female and male Siberian ibex, which represents the estimates of the relative contributions of all the habitat variables to the Maximum entropy model. Among those variables, TRI, DTR, Elevation and Aspect were important characteristics of habitat selection in female Siberian ibex, with TRI (39.4%) the most important (Table 1). Elevation and Aspect were important for habitat selection in male Siberian ibex, with Elevation (62.5%) the most important factor governing their distribution (Table 1).

**Table 1:** Importance of the habitat variables to the Maximum entropy model. The values of importance was normalized to percentages.

Variable	Females	Males
Elevation	19.3	62.5
Aspect	13.3	15.8
Terrain ruggedness index	39.4	9.2
Topographic position index	0.4	2.8
Distance to river	25.5	3.2
Vegetation types	0.7	4.6
Distance to escape terrain	1.5	1.9

### Different environmental requirements of female and male

**Figure 2:**
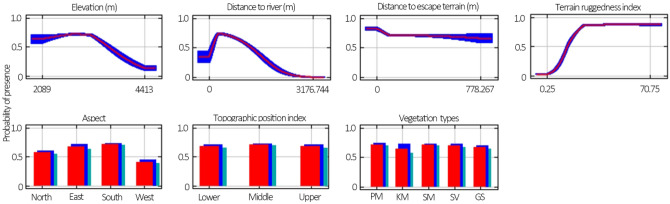
Response curves for the seven variables of females. The curves show the mean response of the 10 replicate Maxent runs (red) and the mean ± one standard deviation (blue, two shades for categorical variables).

**Figure 3:**
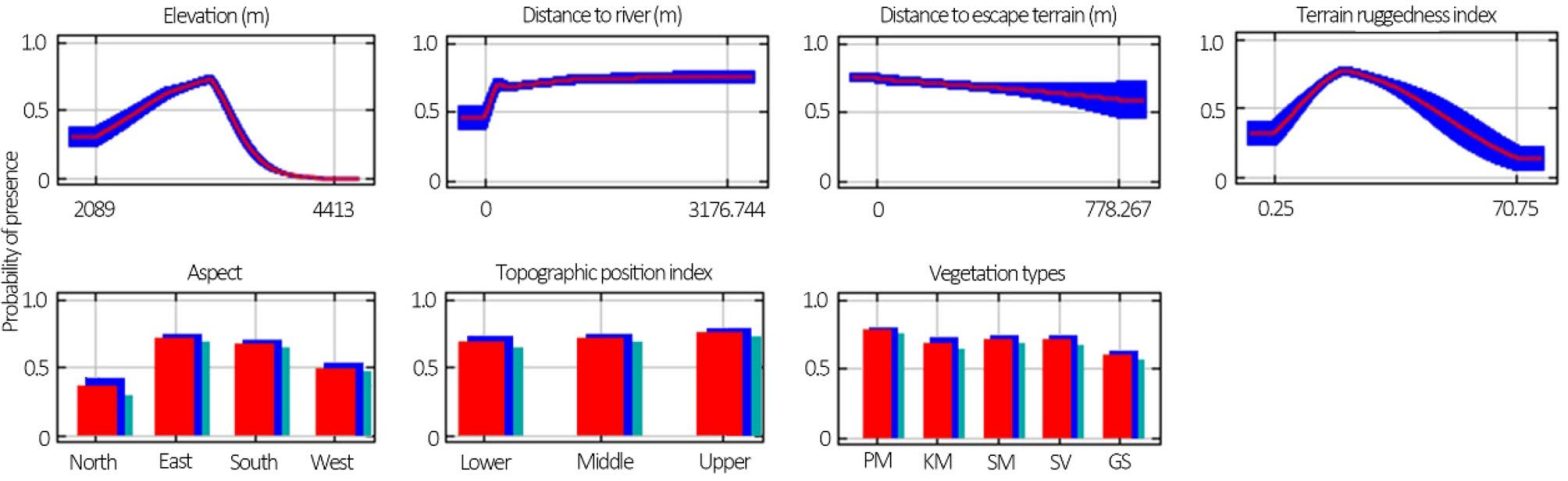
Response curves for the seven variables of males. The curves show the mean response of the 10 replicate Maxent runs (red) and the mean ± one standard deviation (blue, two shades for categorical variables).

We also analyzed the difference in relationships between the habitat suitability and the values of each of the seven habitat variables for both females and males. Both sexes preferred the southern and eastern aspects in summer (Figs. 2, 3), in where the NDVI (southern aspect: 0.224; eastern aspect: 0.228) were higher than that of the western (0.027) and northern (0.176) aspects. The suitable elevations were wider for females (2152 to 3845 m above sea level) than for males (2503 m to 3511 m above sea level), and the mean elevation for females (3001 m above sea level) were lower than for males (3069 m above sea level). Females had a shorter DTR than males, 60 to 1452 m and 60 to 2003 m, respectively (Figs. 2, 3). Females preferred the most rugged terrain (TRI ranged from 13 to 85), and habitat suitability increased with the TRI, but male Siberian ibex preferred a more intermediate rugged terrain (TRI ranged from 3 to 44) as habitat suitability increased with TRI first and then gradually decreased (Figs. 2, 3). The DTET, TPI and VT did not affect the different habitat selections between females and males (Figs. 2, 3).

## Discussion

As expected for any highly dimorphic ungulate, we found that Siberian ibex males and females showed a difference in habitat preference. A similar phenomenon was found also in other ungulates such as the Mediterranean mouflon (*Ovis gmelini musimon*)^7^, red deer^6^, and African buffalo (*Syncerus caffer*)^25^. In general, TRI, DTR, Elevation and Aspect were important habitat resources to female Siberian ibex, while Elevation and Aspect were more important to males. We found that both males and females preferred the southern and eastern aspects, this result may be affected by the forage biomass in different aspects. It has been suggested that, in temperate systems, the NDVI is positively associated with forage biomass over the duration of the growing season^26,27^, as the of southern and eastern aspects had higher NDVI than western and northern aspects, males and females selected southern and eastern aspects would have chance to access better forage. However, males and females had different responses to other environmental factors: (1) females preferred more rugged terrain while males stayed more often in intermediate rugged terrain, (2) females used a wider range of elevations, but males were constrained more in higher elevations, (3) females preferred the sites with a shorter DTR than males.

Similar to our findings, females of other species from the *Capra* genus also preferred more rugged terrain compared to males, for example, the Alpine ibex^22,28^, Nubian ibex^29^, and markhor (*Capra falconeri*)^4^. In sexually dimorphic species, the sexes have large differences in body size and the smaller-sized females have more difficulty escaping predators, making them more vulnerable to predation^30^. In addition, females are often accompanied by their young, which have limited mobility and insufficient predator experience, giving them less chance to escape predators; and predators often selectively target offspring^31^. Females, therefore, may selected greater safety in the more rugged habitats in response to their higher predation risk; in our study area, these areas were small and fragmented. In contrast, males may preferred areas with greater amounts of high-quality food to accumulate energy and increase their body mass as quickly as possible, because their body mass was often the main determining factor in winning male-male contests during the mating season^14^, as a result, we found that males stayed longer in habitat with a lower degree of ruggedness.

Our results also showed that habitat suitability for Siberian ibex females increased with the TRI, indicating that females preferred the most rugged terrain. This result was different from observations of female Dall’s sheep (*Ovis dalli dalli*) that favored an intermediate rugged terrain, possibly because it not only provided sufficient safety against predators, but also a more maneuverable topography that could be important for young^3^. This difference in the degree of preferred ruggedness by these two species may be caused by the different abilities in mobility in rugged terrain of the main predators for each species. The main predators of Dall’s sheep were gray wolves (*Canis lupus*) and coyotes (*Canis latrans*)^32^, while the main predator of the Siberian ibex in our study area was the snow leopard, which has much more mobility in rugged terrain than canids^18,33^. As a result, females of Siberian ibex selected the most rugged areas offering their young the highest chances to evade snow leopards. Many studies also showed that encounters and attacks by predators near cliffs and steep slopes are less likely to end in catching the mountain ungulates^14, 34^.

Rugged terrain has more rocks and a smaller quantity of forage compared to flat meadows^28^, thus less rugged terrain contains more quantity and/or quality of forage. Although the gathering of Siberian ibex females in rugged terrain decreases their access to high quality forage to some extent, they may use a wider range of elevations than males to search for newly emerged and higher-quality vegetation to satisfy their needs for higher nutritional forage. It is well known that newly emerged vegetation has the highest quality nutritional content, with the highest protein and lowest fiber matter^12^. In our study area, as the common vegetation mass increased during summer, plant food quality gradually decreased with a drastic increase in fiber content. Thus, forage of high nutrient value decreased and became more scattered during summer, so dispersing to a wider range of elevations would help females to satisfy their higher requirements for nutrition.

As mentioned above, forage quantity has positive correlation with NDVI, and our result indicated that NDVI had a high negative correlation with elevation, thus in the higher elevations of the TianShan Mountains would possess less quantity forage. However, a narrower range of elevations and higher altitudes that male Siberian ibex generally used, would prevent them from feeding more quantity forage. This phenomenon was contrary to our prediction that Siberian ibex males would prefer habitat with more abundant food, and also contrary to the findings in other ungulates species, such as the mule deer (*Odocoileus hemionus*)^35^, Roosevelt elk (*Cervus elaphus roosevelti*)^36^, and Alpine ibex^22^. Therefore, instead of the differences of food requirements between the sexes in Siberian ibex, we suggested that differences in thermal sensitivity could explain why males preferred the higher elevations.

Previous studies suggested that body size could influence the thermal sensitivity of ungulates, as large bodied individuals have a lower surface area to volume ratio than smaller ones, and therefore had a lower heat dissipation capacity^17^. As a result, in our research, we hypothesized the larger males would prefer the habitat with lower temperatures compared to the smaller females. In addition, pelage color could influence the ability to gain or lose heat: dark-colored animals usually absorb more solar radiation than light-colored animals^37–39^. During the summer, the pelage color of Siberian ibex females is lighter than in males^18^, meaning males would suffer a higher solar heat gain. Generally, the temperature decreases by about 0.5 to 1.0 °C for every 100 m increase in elevation^40^. Therefore, Siberian ibex males, with a lower resistance to high temperatures, would select higher elevations for more comfortable conditions, where stronger wind also reduces the ambient temperature^41^. This difference in thermal sensitivity caused by the difference in body size can also be found in males of the Alpine ibex, where the larger individuals performed higher elevation migrations than smaller individuals in the hot temperatures of summer^41^, and in buffer mouflon, that males preferred habitat where could provide thermal cover than females in hot conditions^42^.

In addition, although the lower thermal sensitivity would prevent Siberian ibex males feeding on pastures with higher biomass, they may adjust their feeding behavior to meet their higher energy requirement. During hot and sunny days, the temperature was low at the beginning and end of daytime, males fed on slopes at lower altitudes, and moved to higher altitudes for resting from late morning to late afternoon or early evening when temperature was high, at this time, almost all males stopped feeding^18^. Therefore, it was not surprised that we found males of Siberian ibex prefer higher elevations where pastures contain lower biomass forage in this study, but males had higher biomass requirement in our previous study^24^.

An alternative hypothesis may be that higher elevation areas are selected by Siberian ibex males to avoid biting insects^18^. Larger hosts often attract more insects as they produce more visual, olfactory and thermal cues that are used by flying insects searching for hosts^43,44^—for example in mixed groups of African cows, tsetse flies (*Glossina* spp.) bit the largest individuals more often than the smaller ones^45^. Therefore, we considered that Siberian ibex males may suffer more from biting insects than the smaller females because of their larger body size. To avoid the biting insects and reduce the costs related to insect bites, male Siberian ibex could demonstrate stronger evasive behavior from flying insects than females by preferring areas with less insects. Anderson et al. reported that stronger winds reduce the activity of insects, therefore the stronger winds at the higher elevations would make the area more suitable for males^46^.

Bleich et al. found that females of the desert bighorn sheep (*Ovis canadensis nelsoni*) were found closer to permanent sources of water^14^. We also found that Siberian ibex females preferred habitat with a shorter DTR than males. Siberian ibex would not drink for long intervals when the liquid content in vegetation is high and dew is present, therefore, the habitat selection by males may be less effected by DTR. But some studies showed that the rate of water loss in larger males was lower than in smaller females^47^, and water requirements of lactating females were higher than for males^48^. Thus a shorter DTR of females may made it be more convenient for them to reach water. Zhu similarly found that Siberian ibex females preferred forage with a higher water content^49^, which also indicates females have a stronger association with water than males.

In conclusion, though multiple factors determine habitat selection in Siberian ibex, only a limited number of key factors may determine their actual distribution. Siberian ibex males and females have different habitat requirements, with Elevation playing a primary role in habitat selection of males, while the TRI is most important for females. Understanding the differences in habitat preference between the sexes has valuable implications for management and conservation efforts for this species.

## Methods and materials

### Study area

This study was conducted in the Eastern TianShan Mountains, Xinjiang, China (43°2′25″–43°13′59″ N, 86°47′40″–87°10′12″ E). The total study area is 307.67 km^2^ and contains rugged ridges amid a complex of narrow and wide valleys. Elevations range between 2089 and 4413 m above sea level. This region has semi-humid to semi-arid transition zones with a temperate continental climate, making local conditions cold and arid, typical for the entire Eastern Chinese TianShan Mountains^50^. The annual average temperature is − 1.0 °C, with an extreme high temperature of + 30.5 °C, which is common for July, and an extreme low temperature of − 30.2 °C, which is observed in January. Cyperaceae and Poaceae are relatively dominant in the local plant community, with a mixture of other families, such as Polygonaceae, Asteraceae, Fabaceae, Ranunculaceae, and Rosaceae^50^. Siberian ibex and red deer (*Cervus elaphus*) are common ungulates in this region, along with predators such as snow leopards (*Panthera uncia*) and wolves (*Canis lupus*); raptors, such as the cinereous vultures (*Aegypius monachus*), bearded vultures (*Gypaetus barbatus*) and golden eagles (*Aquila chrysaetos*) can also be found here^50^.

### Maximum entropy model

In recent years, a large and growing body of applications of species’ distribution models (SDMs) have been used to investigate ecological and biogeographical issues, for example, assess habitat suitability^20^, identify potential ecological corridors^51^, and predict suitable habitats for species reintroduction^52^. The Maximum entropy model is one of the most widely used models of SDMs^53^. With presence-only distribution data of species and the environmental characteristics of those locations, the Maximum entropy model can predict a species’ distribution and identify the key factors that affect that distribution to a high degree of accuracy by employing a “maximum likelihood method”^53^. The Maximum entropy model is effective in dealing with small sample sizes and has a more accurate performance compared to other models^54^. Therefore, we chose to use Maximum entropymodel to research the difference of habitat selection by Siberian ibex males and females, considering the relatively limited occurrence data.

### Transect surveys

Our census was conducted monthly during summer (June to August) from 2016 to 2018. We traveled by car and on foot within the same area and along the same route. We started routes at valley entrances and conducted our survey along the valley floors; the transects covered the entire valley floor. Along the transect line, we stopped every 2–3 km and searched for ibex using binoculars (magnification 8 ×) and a telescope (magnification 20–60 ×). To avoid the possible disturbance of observed animals, the observations were done from a distance of usually more than 200 m. During data collection, we noted the following information: date, and sex and age of each individual. We recorded all females (adult or sub-adult) and males, identifying the age of males by counting horn annuli when visible. We also recorded the GPS of observers, the distance and angle between observers and Siberian ibex by using the range finder and compass, based on these information, the geographical position of Siberian ibex was obtained, and then located on a 1:50,000 topographical map during field work. In addition, the topographical map was also helpful to increased the accuracy of geographical position of Siberian ibex.

### Mapping survey data

**Figure 4:**
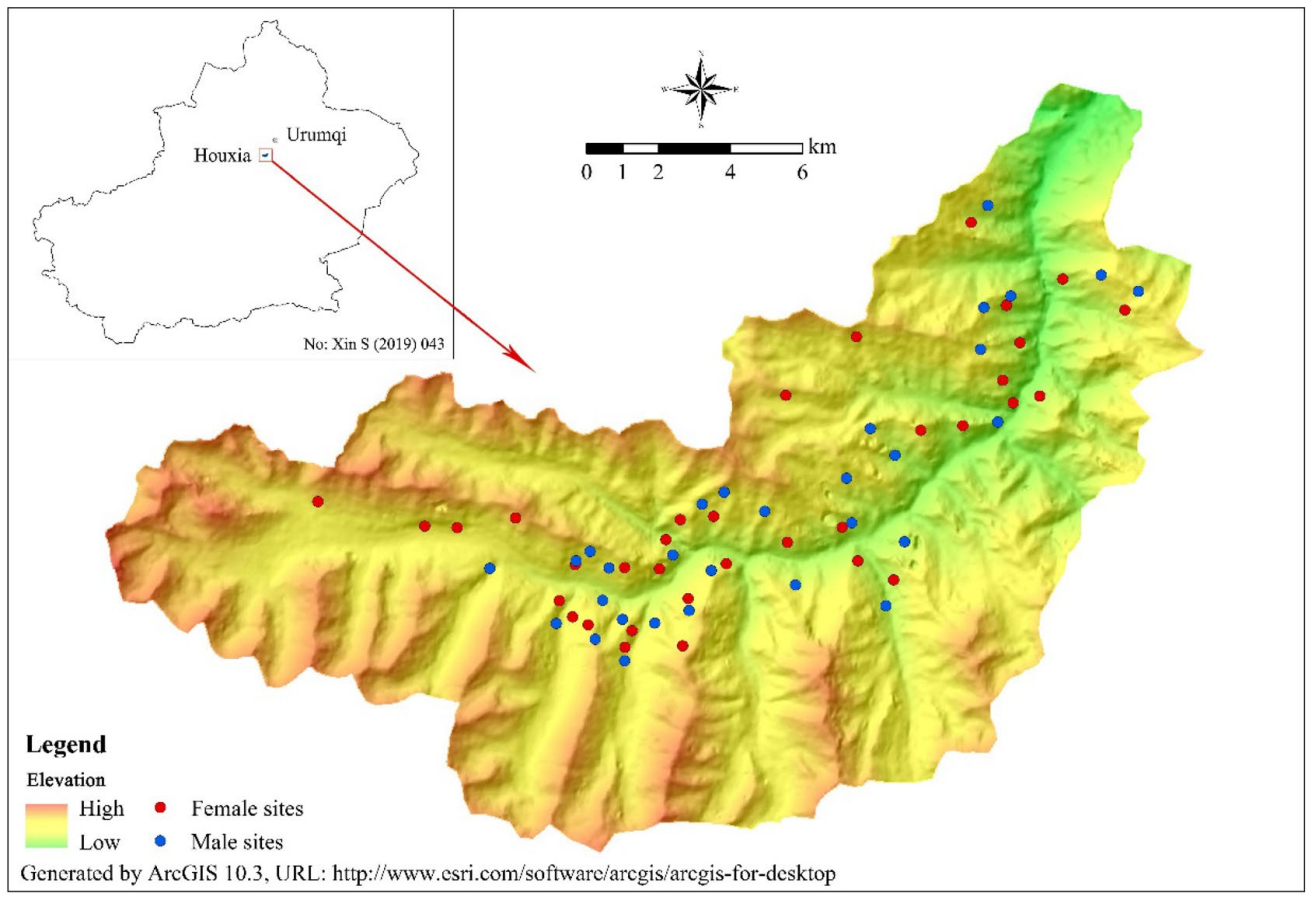
Study area in the Eastern TianShan Mountains, Xinjiang, China and distribution of female and male sites.

The female and male location data was processed and converted into GIS layer (30 × 30 m) grid cells showing the species’ location using a projected coordinate system (UTM 45 N) with WGS 1984 projection for the purpose of data compatibility. For each sex, in order to avoid the effect of overfitting due to the spatial autocorrelation of the Siberian ibex occurrence data, we used the Buffer analysis to selected field data, the buffer radius was set as 0.25 km, deleted the location data randomly until the distance between all of the rest location points were more than 0.5 km^55^. Finally, 34 female and 30 male point locations on 30 × 30 m grid cells were collected, and were shown in the elevation map of study area (Fig. 4).

### Habitat variables

Habitat variables are as follow: Elevation, Slope, Aspect, Topographic position index (TPI), Terrain ruggedness index (TRI), Distance to river (DTR), Distance to escape terrain (DTET), Vegetation types (VT), and Normalized difference vegetation index (NDVI).

We downloaded the digital elevation model (DEM) data with 30 m spatial resolution from the geospatial data cloud platform of the computer network information center of the Chinese Academy of Sciences (http://www.gscloud.cn). Elevation was extracted directly from DEM. Slope and Aspect were calculated using a surface tool in ArcGIS’s Spatial Analyst Tool^56^. Aspect was categorized into four classes: north (315°–45°), east (45°–135°), south (135°–225°) and west (226°–315°). TPI was calculated using the Land Facet Corridor Designer in ArcGIS, and was categorized into three classes: upper, middle and lower. TRI shows the average change in elevation between a center pixel and its eight neighboring pixels in a 3 by 3 window, and it was calculated from the DEM based on the work of Sappington et al.^57^. River data was downloaded from the Standard map of the natural resources department of the Xinjiang Uygur Autonomous Region (http://xj.tianditu.gov.cn/main/bzdt.html). DTR was calculated using Euclidean Distance in the Spatial Analyst Tool. We extracted the ridges from DEM with Arc Hydro Tools, and overlapped the ridges and steep slopes (> 45 degree) to produce escape terrain, then compute the DTET with ArcSpatial Analyst tools^20^. VT data were obtained from 1:1 000 000 vegetation types map of China, it was categorized into five classes: *Poa* spp. alpine meadow (PM), *Kobresia capillifolia* alpine meadow (KM), *Kobresia stenocarpa* alpine meadow (SM), *Saussurea* spp., *Rhodiola rosea*, *Cremanthodium* spp. sparse vegetation (SV), and glacier and snow cover (GS). NDVI was used to characterize relative primary productivity of forage quantity^26^. We first downloaded the remote sensing image data at 30 m resolution, which imaging time was in July, 2018, from the United States Geological Survey (USGS) (http://glovis.usgs.gov/), then the data was preprocessed with wave band synthesis, radiometric correction, atmospheric correction and clipping by ENVI 5.3, finally we obtained the NDVI data. All those data were converted to ASCII files.

### Mapping suitable habitat for males and females

We used the Maxent program MaxEnt 3.3.3 k (http://www.cs.princeton.edu/-schapire/MaxEnt/) as described in detail in Phillips and Dudik^53^. For the Maxent analysis, we used a Java environment. We used the following settings of MaxEnt: automatic feature selection, regularization multiplier at unity; max number of background points was set to 10 000, 10 replicates. We also used a random test percentage of 25%. Maps of the potential distribution of the species using the logistic output format were produced, which is the easiest output to conceptualize and which provides an estimate of probability of presence between zero and one^53^. A jackknife test was used on the AUC (area under the curve) to determine the weight of each variable within each model.

We first ran the Maximum entropy model with all habitat variables to obtain the importance of each variable, then used the "Band Collection Statistics" of Spatial Analyst Tools in ArcGIS to conduct correlation analysis on those habitat variables in order to avoid interference with model analysis results due to high autocorrelation between habitat variables. When two or more variables had a correlation coefficient > 0.75 or < − 0.75, we deleted the habitat variable that was least important. We repeated this step until all the correlation coefficients were < 0.75 or < − 0.75. Finally, Slope and NDVI were deleted in the final model, as Slope had a high positive correlation with TRI, and NDVI had a high negative correlation with Elevation.

Model fit was measured using the AUC from the receiver operating characteristic curve, which is the relationship between the sensitivity and the false positive fraction, with a value of 0.5 representing a random model^58^. The quality of model outputs is decided by the threshold of AUC: > 0.9 is excellent, 0.8 to 0.9 is good, 0.7 to 0.8 is fair, 0.6 to 0.7 is poor, and < 0.6 is fail^59^. The habitat suitability maps predicted by the MaxEnt model was reclassified according to the MST (maximized sum threshold) criteria^60^. The area with suitable values that was larger than what was indicated by the MST criteria would reclass to suitable, otherwise, unsuitable, after that, the suitability maps for males and females were obtained.

To showed the different habitat preference by male and female Siberian ibex, we used 1 and 0 to represented the suitable and unsuitable habitat for both males and females, then using the Map Algebra tool in ArcGIS’s Spatial Analyst Tool, we calculated the distribution of suitable and unsuitable habitat for males and females as follows: Y = Males × 10 + Females, where Males and Females were the suitability maps for males and females, respectively; Y equaled to 0 meant the area was unsuitable for both females and males (*A*_*non*_), Y equaled to 1 meant the area is only suitable for females (*A*_*f*_), Y equaled to 10 meant the area was only suitable for males (*A*_*m*_), Y equaled to 11 meant the area was suitable for both females and males (*A*_*fm*_). We also calculated the overlap rate of females’ and males’ suitable habitat, with the following equation: overlap rate = *A*_*fm*_/ (*A*_*f*_ + *A*_*m*_). In addition, the suitable habitat fragmentation of male and female Siberian ibex was calculated by using the mean size of the patches (A) as following equation: *A* = *A*_*Li*_/*N*_*i*_, where *A*_*Li*_ was the total suitable habitat area of species *i*, *N*_*i*_ was the number of suitable habitat patches of species *i*, smaller *A* meant the more fragment of suitable habitat^61^.

### Ethical approval

No further approval by an Ethics Committee was required, as behavioural observations at a distance in this study were non-invasive.

## Supplementary Information

Supplementary Information.

## Data Availability

The datasets generated and/or analyzed during the current study are available from the corresponding author on reasonable request.
